# Synthetic HIV-1 matrix protein p17-based AT20-KLH therapeutic immunization in HIV-1-infected patients receiving antiretroviral treatment: A phase I safety and immunogenicity study

**DOI:** 10.1186/1471-2334-14-S2-O6

**Published:** 2014-05-23

**Authors:** Arnaldo Caruso, Marialuisa Iaria, Francesca Caccuri, Cinzia Giagulli, Simona Fiorentini

**Affiliations:** 1 University of Brescia, Brescia, Italy

## Introduction

Therapeutic vaccination is a promising approach to treat HIV-1+ people by boosting or redirecting immune system to neutralize HIV-1 antigens whose effects are relevant to viral pathogenesis. HIV-1 matrix protein p17 is a structural protein that, through the interaction of its NH2-residing epitope AT20 with specific receptor(s), acts extracellularly as a viral toxin. In fact, p17 is able to deregulate biological activities of different cells involved in AIDS pathogenesis.

To induce neutralizing antibodies (Abs) to p17 we developed a peptide-based immunogen (AT20-KLH) and evaluated its safety and immunogenicity.

## Methods

24 asymptomatic HAART-treated HIV-1+ patients were enrolled in a phase I clinical study and were randomized to 3 groups: 2 groups treated with five IM injection (Arm A: 25 μg/inoculation; Arm B: 100 μg/inoculation) at day 0, 28, 56, 84 and 112; the control group (Arm C), not injected. Safety was assessed by monitoring local and systemic adverse events (AEs), recorded till day 168. Evaluation of immunogenicity was by titering Abs using ELISA.

## Results

In all, 105 AEs were reported across the groups. Most were mild and resolved without sequelae. No significant changes in the routine laboratory parameters, CD4 T-cell count or HIV-1 viremia were found. Despite the absence of anti-AT20 preimmunization Abs, 100% of treated subjects developed high titers of anti-AT20 Abs (GM 9775) in response to both AT20-KLH doses. These Abs were also capable of recognizing AT20 within the p17 framework and to neutralize its binding to cell receptors. Furthermore, we were able to recruit several participants two years after the first injection to evaluate longevity of anti-AT20 elicited Abs. 100% of these subjects still showed anti-AT20 Abs with titers that were about half the ones detected at the end of immunization protocol.

## Conclusion

The AT20 peptide-based approach has allowed to redirect HAART-treated patients’ humoral responses toward a previously untargeted hotspot of functional activity. Overall, the tested AT20-KLH doses were safe and well tolerated, supporting further exploration of AT20-KLH as an HIV-1 therapeutic vaccine candidate.

